# Real-Time Pattern-Recognition of GPR Images with YOLO v3 Implemented by Tensorflow

**DOI:** 10.3390/s20226476

**Published:** 2020-11-12

**Authors:** Yuanhong Li, Zuoxi Zhao, Yangfan Luo, Zhi Qiu

**Affiliations:** 1 College of Engineering, South China Agricultural University, Guangzhou 510642, China; liyuanhong@stu.scau.edu.cn (Y.L.); luoyangfan@stu.scau.edu.cn (Y.L.); qiuzhi@stu.scau.edu.cn (Z.Q.); 2 Ministry of Education Key Technologies and Equipment Laboratory of Agricultural Machinery and Equipment in South China, South China Agricultural University, Guangzhou 510642, China; 3 Department of Biological and Agricultural Engineering, Texas A&M University, College Station, TX 77843, USA

**Keywords:** pattern-recognition, real-time, YOLO v3, TensorFlow, electromagnetic wave

## Abstract

Artificial intelligence (AI) is widely used in pattern recognition and positioning. In most of the geological exploration applications, it needs to locate and identify underground objects according to electromagnetic wave characteristics from the ground-penetrating radar (GPR) images. Currently, a few robust AI approach can detect targets by real-time with high precision or automation for GPR images recognition. This paper proposes an approach that can be used to identify parabolic targets with different sizes and underground soil or concrete structure voids based on you only look once (YOLO) v3. With the TensorFlow 1.13.0 developed by Google, we construct YOLO v3 neural network to realize real-time pattern recognition of GPR images. We propose the specific coding method for the GPR image samples in Yolo V3 to improve the prediction accuracy of bounding boxes. At the same time, K-means algorithm is also applied to select anchor boxes to improve the accuracy of positioning hyperbolic vertex. For some instances electromagnetic-vacillated signals may occur, which refers to multiple parabolic electromagnetic waves formed by strong conductive objects among soils or overlapping waveforms. This paper deals with the vacillating signal similarity intersection over union (IoU) (V-IoU) methods. Experimental result shows that the V-IoU combined with non-maximum suppression (NMS) can accurately frame targets in GPR image and reduce the misidentified boxes as well. Compared with the single shot multi-box detector (SSD), YOLO v2, and Faster-RCNN, the V-IoU YOLO v3 shows its superior performance even when implemented by CPU. It can meet the real-time output requirements by an average 12 fps detected speed. In summary, this paper proposes a simple and high-precision real-time pattern recognition method for GPR imagery, and promoted the application of artificial intelligence or deep learning in the field of the geophysical science.

## 1. Introduction

In the application of ground-penetrating radar (GPR) engineering detection, the following three cases are the most common: (1) Inspection of the atypical situation of reinforced concrete structures such as bridges, tunnels, or public roads, or the number of steel bars inside those structures; (2) locating certain objects underground, such as archaeological research; (3) evaluating and measuring the distribution of hollows, voids, or soil firmness in highways, bridges, and tunnels. Nonetheless, the outcomes, after GPR detection, are often judged by the worker’s experience to recognize the location and size information of the target [1,2]. Actually, these kinds of evaluations using GPR image are not infeasible, but consume a lot of manpower and material resources. For example, the 3D radar launched currently by MALA can collect data on multiple channels. Suppose that a 3D GPR with 22 acquisition antennas can generate 22 GPR images at the same time; if we evaluate its image outcome by traditional method, the analysis work will be very inefficient [3,4]. Besides, with the continuous development of 3D radar imaging technology, especially for multi-channel GPR, efficient and intelligent AI algorithms can not only output the analyzed results automatically, but also can fulfill the application demands of the underground exploration engineering, such as long-distance detection of reinforced concrete of roads; investigation of large-area cavities of bridges and tunnels; early warning of urban road collapse.

In terms of using artificial intelligence to identify GPR imagery, Sonoda and Kimoto (2018) adapted the finite-difference time-domain (FDTD) to simulate multiple GPR images, and trained a 9-layer deep neural network (DNN) model to extract feature maps that contain many hyperbolic signals of underground objects [5]. Finally, they obtained the characteristics of electromagnetic wave intensity from the curve signal and identified six materials with 80% accuracy. Because of the limited quantity of DNN layers, this method lacked accuracy of the identified materials and is limited to its sample selection. Aydin and Yüksel (2017) adapted the GprMax simulation API program to generate GPR B-scan images, then proposed to combine two convolutional layers and pooling layers to classify the electromagnetic wave, but they did not involve in-depth or improved research in classified speed [6]. Dinh et al., (2018) validated performance with traditional GPR images processing algorithms and convolutional neural network (CNN). At last, the reinforcement in GPR images were positioned and inspected automatically. After analyzing 26 bridge decks GPR data, they achieved a recognition accuracy of 99.60% ± 0.85%; but its detecting speed did not fulfill the engineering demands of real-time outcome [7]. Pham and Lefèvre (2018) used the faster-RCNN framework to detect hyperbola reflections from many B-Scans generated from gprMax toolbox and the results show that faster-RCNN framework can provide significant improvements to deal with GPR data [8,9,10,11]. Kechagias-Stamati et al. proposed a CMNet network for synthetic aperture radar (SAR) image target recognition based on convolutional neural network. The network adds center loss and softmax training process to the feature layer of SAR images [12]. In order to improve the target recognition rate of SAR image, both intra class aggregation and inter class separation were considered. However, this significantly reduces the utilization of hyperbolic features in GPR images. Dou et al. (2017) proposed a novel technique called column-connection clustering (C3) algorithm to separate hyperbolae in GPR images, and obtain hyperbolic signatures. This method can also be used for real-time detection [13]. The fitting speed is 0.73 s per hyperbola. However, the number of hyperbolic objects in GPR images is often large. Compared with the Yolo V3 recognition method proposed in this paper, the recognition speed of 12 frames per second per image is more dominant. In addition, they only test the scattered hyperbola. On this basis, this paper also tests the hyperbola-intensive samples, and achieves ideal detection effect. Pham et al. (2020) proposed an improved YOLO structure called YOLO-fine to detect very small objects from aerial and satellite remote sensing images. However, for GPR images, we not only need to detect small objects, but also need to identify intensive hyperbolic features [14]. Obviously, in many engineering cases, very small hyperbolic features are not the common situations.

## 2. Materials and Methods

### 2.1. YOLO v3 Feature Extractor

**Figure 1:**
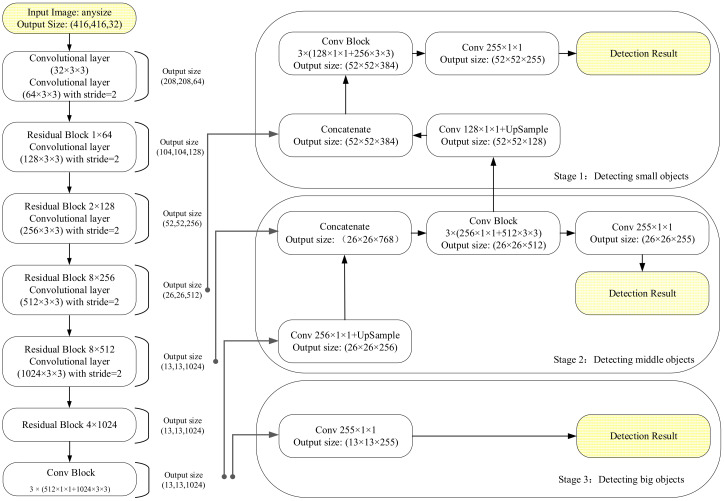
You only look once (YOLO) v3 architecture.

YOLO v3 is a classical pattern-recognition algorithm based on darknet-53 CNN architecture proposed by Joseph Redmon in 2018 [15]. It is currently a marketable object detection algorithm. Most importantly, it has ultra-fast detected speed than SSD, but almost as accurate as faster-rcnn [16,17]. The YOLO v3 basic framework contains the convolutional layer, batch normalization (BN) layer, and leaky rectified linear unit (ReLU) layer [18,19]. First, it is assumed that all input images are resized into 416 × 416 and three types of feature maps; then they go through a 32 × 3 × 3 (filter numbers are 32 with 3 × 3 sizes) and 64 × 3 × 3 convolutional layer, and output a 208 × 208 feature map with 64 channels. Where the second block network of YOLO v3 carried one residual block which includes zero padding, convolution and residual unit, and 128 × 3 × 3 convolutional layer; it outputs some 104 × 104 feature maps with 128 channels. The third YOLO v3 network contains two residual block, then go through 256 × 3 × 3 convolutional layer, where the 4th block contains many residual shortcut to all 256 × 256 feature maps. In addition, this block makes vector concatenated operation of residual shortcut to reduce the gradient explosion and outputs 52 × 52 feature maps with 384 channels. With up-sample, some 52 × 52 feature maps are outputted for YOLO v3 to detect small-scale objects [20]. Similarly, the fifth block outputs many 26 × 26 feature maps for detecting medium-scale targets. At last, network still passes by many residual shortcut connection blocks which include zero padding, convolution, and residual unit. Finally, YOLO v3 designed a 255 × 1 × 1 convolution layer to output 13 × 13 feature maps with 255 channels for detecting big objects [21]. In general, YOLO v3 can detect images on three different scales with 32 × 32, 16 × 16, and 8 × 8 feature maps, where the first detected operation layer is at 82th layer; its stride takes 32 to generate 13 × 13 feature maps. The second up-sampling operation is at 94th and the third detection layer is at 106th layer, which produces a feature map with dimensions 52 × 52 × 255. Overall architecture of YOLO v3 is shown in Figure 1. In addition, we used the K-means clustering to select bounding box priors in YOLO v3.

**Figure 2:**
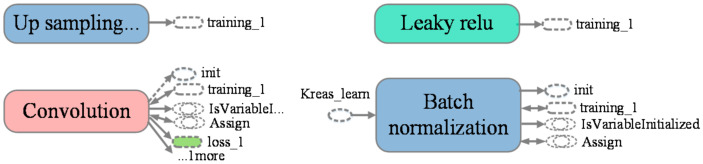
YOLO v3 visual format in TensorBoard.

The following Figure 2 shows a part graph of YOLO v3 exported from TensorBoard of TensorFlow visualization API, which was actually a neural network connection diagram for YOLO’s second up sampling. The TensorBoard can show the output and input tensor variables at each node, in addition, it can show the dependency between the tensor operations through some edges. The conv2d here is abbreviated for the convolution layer or block in Figure 1. Similarly, Leaky relu is denoted as ReLu layers in Figure 1 and batch normalization is denoted as BN layer. We can visualize all concatenation operation of each stage of YOLO v3 through TensorBoard, such as the attributes behind the convolution layer indicate that the input and output tensors correspond to this convolutional layer. The loss represents the value of the current convolutional layer after passing through the optimizer.

### 2.2. Bounding Box Encoding Strategy

**Figure 3:**
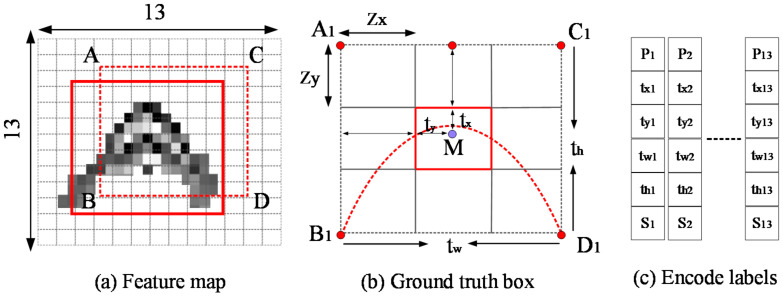
Improved YOLO v3 bounding box encoding: (**a**) Feature map of GPR target in YOLO v3; (**b**) Ground truth box of hyperbolic signal; (**c**) Encoding format of detecting GPR target.

Soil objects underground are regularly sensitive to electromagnetic waves caused by its physical properties [22,23]. Most of them appear as parabolic with openings downward or obvious energy reflection in electromagnetic waves format. GPR moves along the survey line and continuously collects a series of trajectories (A-scan) to form electromagnetic wave B-scan images [24]. Before YOLO v3 training, GPR images were collected in this way. As mentioned in Section 2.1, YOLO v3 outputs feature maps or cells by three different stages, and each bounding box is responsible for multiple categories [25]. Suppose that the input GPR images size still is 416 × 416, as shown in Figure 3a, then the original picture can be divided into 13 × 13 cells. Those cells that are parabolic vertex M in the GPR images are responsible for predicting corresponding targets. When annotating GPR image samples, we make the center of ground truth box (rectangle A1B1C1D1) to correspond to the position of parabola apex. Red box in Figure 3a contains the midpoint of target wave; rectangle A1B1C1D1 marked as red solid line is ground truth bounding box and ABCD marked as red dotted line represents the predicted box. Figure 3b shows the encode ways of ground truth box in YOLO v3. Point A_1_ is the top left corner of box; t_x_ and t_y_ are the pixel position of point A_1_ in GPR image. Zx and Zy are noted as pixel width and height of each cell respectively. The width of B_1_D_1_ is marked as t_w_ and the height of C_1_D_1_ is marked as t_h_. As shown in Figure 3c, each bounding box was attributed to one object score or confidence$\;P_{i}\in \left\{0,1\right\}$, 4 box coordinates (t_x_, t_y_, t_w_, t_h_), and one class score S_i_. Here, S_i_ follows similarly as the one hot encoding method and$\;S_{i}\in \left[0,1\right]$. If S_i_ equals to 0, there was no current detected target in the GPR image; otherwise, if S_i_ equals to 1, it indicates that there exists current detected target. Finally, the feature map corresponds to 13 × 13 cells, and the output bounding box encoded tensor shape is (13 × 13, 6). If there were n GPR image samples, then all bounding box sizes corresponded to tensor (n, 13 × 13, 6). It is worth noting that if it is a non-hyperbolic target, such as the voids detected below, we will use the original encoding method of YOLO v3.

### 2.3. Anchor Box Selection by K-Means Clustering

The K-means is an iterative algorithm that can divide data into K predefined clustering and cluster each point into specific data groups [26]. When YOLO v3 trains GPR sample data, anchor box can control skillfully the over fit recognition results of soil targets, because in the high-frequency electromagnetic wave reflection signal, when two target positions are relatively close, those close parabolic vertices will be easily assigned to the same bounding box. K-means defines the size of bounding box through cluster analysis. Absolutely, K-means tries to keep clusters as different as possible at this point in order to minimize the sum of squared distances between all centers of data clusters [27,28]. First, we define K value and initialize the centroids by shuffling, then keeping iterating until there is no change in the centroids outcome. This is called expectation maximization [29]. Assuming that there are m samples, here we introduce a multi-sample function about K value:

(1) $$F=\;\overset{m}{\underset{i=1}{\sum }}\overset{k}{\underset{k=1}{\sum }}\sigma_{ik}∥t\left(x,y\right)-\mu_{k}{∥}^{2}$$

If the point $t\;\left(x,y\right)$ belongs to the K cluster, then$\;\sigma_{ik}=1$ otherwise$\;\sigma_{ik}=0$; at this time, $\mu_{k}$ can be considered as the centroid $\mathrm{of}\;\;t\;\left(x,y\right)$. If the derivative of F function can minimize the equation solution, then the problem can be solved using the following formula:

(2) $$\frac{\partial F}{\partial \sigma_{ik}}=\;\overset{m}{\underset{i=1}{\sum }}\overset{k}{\underset{k=1}{\sum }}∥t\left(x,y\right)-\mu_{k}{∥}^{2}$$

Here, it is needed to distinguish F solution and recalculate the centroid after the last clustering iteration. Obviously, data points $t\;\left(x,y\right)$ are assigned to close clusters. Finally, we can recalculate each cluster centroid according to the following Equation (3) to reflect the situation of the new point allocation.

(3) $$\frac{\partial F}{\partial \mu_{k}}=\;2\overset{m}{\underset{i=1}{\sum }}\sigma_{ik}\left(t\left(x,y\right)-\mu_{k}\right)=0$$

(4) $$\mu_{k}=\;\frac{\sum_{i=1}^{m}\sigma_{ik}t\left(x,y\right)}{\sum_{i=1}^{m}\sigma_{ik}}$$

**Figure 4:**
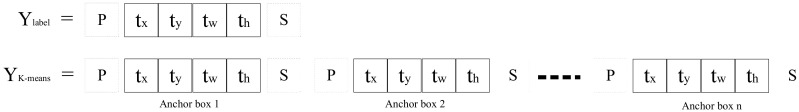
Encoding bounding box with multiple anchor box after k-means selection.

K-means uses data distance as the evaluated criterion to determine the selection of anchor box. Algorithm iteration is initialized at the beginning. In order to avoid the F function staying at the local optimal rather than global optimal, this paper adopts a variety of centroid initialization to run the K-means algorithm [30,31]. After filtering by K-means, the encode label of YOLO v3 for GPR image increases the data dimension. As shown in Figure 4, $Y_{label}$ represents the encoded bounding box without increasing the dimension. This is the transpose of data matrix in Figure 3c. $Y_{K-Means}\;$ represents the encoded bounding box that have added n anchor boxes output by K-means clustering.

### 2.4. Principle Analysis of V-IoU Processing with NMS

Non-maximum suppression (NMS) is commonly applied to extract the window with the highest score in detection algorithm, such as feature extraction in sliding windows, pedestrians in automatic driving, and vehicle recognition [32]. Similarly, in the GPR image, after feature maps are produced by the convolutional layer of YOLO v3 in the 3rd stage recognized by the classifier, for some underground targets, there are a large number of bounding boxes that cross each other or contain the same parabolic midpoint in one cell. The goal of NMS is to remove the detected redundant boxes and keep the best one. First, it is needed to mention here the intersection over union (IoU) score. IoU is a standard performance metric for image category segmentation problems [33]. For a given set from image, IoU defined by Equation (5) gives the ratio of intersection and union of the predicted bounding box and ground truth bounding box [34]. Suppose t represents the probability outputs of pixel set N after filter by activation function in the GPR image; Y denotes the data set composed of ground truth bounding box; $Y\in {\left\{0,1\right\}}^{M}$ marks 0 for non-target pixels and 1 for target pixels.

(5) $$\mathrm{IoU}=\;\frac{I\left(t\right)}{U\left(t\right)}=\frac{\sum_{n\in N}t_{n}*Y_{n}}{\sum_{n\in N}\left(t_{n}+Y_{n}-t_{n}*Y_{n}\right)}$$

First, in YOLO v3, NMS can calculate the confidence C of the proposal region and sort the bounding boxes list. Second, NMS selects the predicted box with the largest score; then the IoU coefficients of other remain bounding boxes and the current box are calculated. If the IoU value is greater than the predefined threshold, NMS will delete this bounding box [35]. This is a complete iterative process in which NMS is applied to select the maximum score bounding box for one target. Then in the second iteration, the highest score box is still selected in the remaining boxes and those that exceed the predefined IoU threshold are deleted until all possible targets in the GPR image have been pick up.

**Figure 5:**
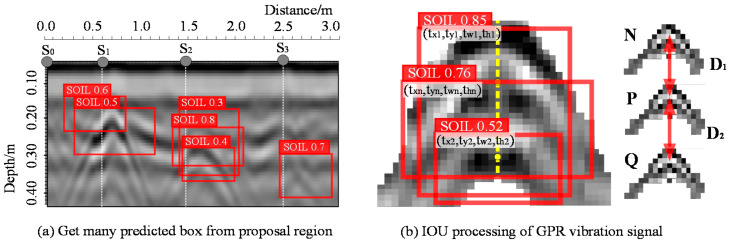
V-IoU principle with non-maximum suppression (NMS): (**a**) The predicted boxes from proposal region; (**b**) The IoU processing of GPR vibration signal.

After the YOLO v3 residual network and 1 × 1 convolutional layer, a large number of bounding boxes are generated on the region proposal area outputted by feature map. As shown in Figure 5a, S_0_ denotes the starting position of GPR: S_1_, S_2_, and S_3_ represent respectively the soil surface position of parabolic electromagnetic wave signal generated by three iron cylinders with buried depths of 0.25 m, 0.3 m, and 0.35 m, respectively. The soil dielectric constant is about 6.5 and the electrical conductivity is about 0.002 s/m. It can be seen that there are numerous prediction bounding boxes around each target. Now we focus on one parabola. In the process of YOLO v3 algorithm recognizing the target from GPR image, it is uncomplicated to misidentify the parabola originally belonging to one object as multiple targets because of the oscillating signal from electromagnetic wave [36]. The points N, P, and Q in Figure 5b represent three parabola generated by some strong conductive targets in depth direction of the soil. The number on a side of SOIL label represents the probability of being identified as a target, with the maximum value as 1 and the minimum as 0. YOLO v3 recognizes or locates those as three adjacent targets, but it is only one target, although their IoU threshold has been included in the predefined range. Therefore, this paper proposes the principle of V-IoU merging vacillate signals of similarity waves based on GPR images. Assume the location of ground truth box (red box) was marked as coordinate (t_xn_, t_yn_, t_wn_, t_hn_) and the locations of another two boxes which were marked by GPR echo signal vacillation were predicted as (t_x1_, t_y1_, t_w1_, t_h1_) and (t_x2_, t_y2_, t_w2_, t_h2_). Then the coordinate of ground truth box of point N can be denoted as$\;\left(t_{xn}+\frac{t_{wn}}{2},t_{yn}-\frac{t_{hn}}{2}\right)$. Similarly, the pixel coordinate of P is denoted as $\left(t_{x1}+\frac{t_{w1}}{2},t_{y1}-\frac{t_{h1}}{2}\right)$ and Q is denoted as$\;\left(t_{x2}+\frac{t_{w2}}{2},t_{y2}-\frac{t_{h2}}{2}\right)$. First, it is worth noting that we define a horizontal threshold β here and make the $\left(t_{xn}-t_{x1}\right)+\left(\frac{t_{wn}}{2}+\frac{t_{w1}}{2}\right)\in \left[-\beta ,\beta \right]$. At the same time we define a longitudinal threshold α and made the$\;\left(t_{yn}-t_{y1}\right)-\left(\frac{t_{hn}}{2}-\frac{t_{h1}}{2}\right)\in \left[-\alpha ,\alpha \right]$; if those parabolic midpoint or N, P, and Q points at the soil depth satisfy the horizontal and vertical critical values, we will liberate the limitation of IoU threshold and merge those prediction boxes. This is the core idea of V-IoU, for example, $D_{i}\in \left[-\alpha ,\alpha \right]$ and i∈R.

### 2.5. Loss Function and Learning Rate Adaptive Optimizer

Loss function of YOLO v3 in this paper is composed of mean variance and error [37]. Specifically, it is mainly divided into three parts for the calculation of offset losses, midpoint coordinate of parabola in GPR image prediction error gprErr, V-IoU prediction error viouErr, and classification error clsErr [38]. Here preset the weight of gprErr $\gamma_{gpr}\;$ as 5 and the weight of viouErr $\gamma_{viou}$ as 0.5 in order to rectify the domination of large target is weaker than the small target during detection. It can be expressed by the following formula:

(6) $$\mathrm{Loss}=\;\overset{s^{2}}{\underset{i=0}{\sum }}gprErr+viouErr+clsErr$$

**Figure 6:**
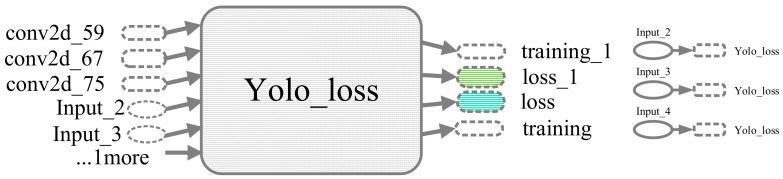
YOLO v3 loss visual format in TensorBoard.

After derivation, the loss function of this three parts can be expressed as:

(7) $$\begin{array}{ll} \mathrm{gprErr}=\gamma_{gpr} & \overset{s^{2}}{\underset{i=0}{\sum }}\overset{B}{\underset{j=0}{\sum }}I_{ij}^{tar}\left[{\left(x_{i}-\hat{x_{l}}\right)}^{2}+{\left(y_{i}-\hat{y_{l}}\right)}^{2}\right]\\ & +\gamma_{gpr}\overset{s^{2}}{\underset{i=0}{\sum }}\overset{B}{\underset{j=0}{\sum }}I_{ij}^{tar}\left[{\left(\sqrt{w_{i}}-\sqrt{\hat{w_{l}}}\right)}^{2}+{\left(\sqrt{h_{i}}-\sqrt{\hat{h_{l}}}\right)}^{2}\right] \end{array}$$

where $\hat{x_{l}}$, $\hat{y_{l}}$, $\hat{w_{l}}$, and $\hat{h_{l}}$ in the Equation (7) are denoted as predicted values by YOLO v3, $x_{i}$, $y_{i}$, $w_{i}$, and $h_{i}$ expressed as training tag value; $I_{ij}^{tar}$ indicates that if the object falls into the j-th position of lattice i-th bounding box, its value is either 1 or 0.

(8) $$\mathrm{viouErr}=\overset{s^{2}}{\underset{i=0}{\sum }}\overset{B}{\underset{j=0}{\sum }}I_{ij}^{tar}{\left(C_{i}-{\hat{C}}_{i}\right)}^{2}+\gamma_{viou}\overset{s^{2}}{\underset{i=0}{\sum }}\overset{B}{\underset{j=0}{\sum }}I_{ij}^{notar}{\left(C_{i}-{\hat{C}}_{i}\right)}^{2}$$

where ${\hat{C}}_{i}$ in the Equation (8) is denoted as predicted value by YOLO v3, $C_{i}$ expressed as training tag value, $I_{ij}^{notar}$ indicates that the j-th bounding box of the object grid *i* does not contain the detection target.

(9) $$\mathrm{clsErr}=\;\sum_{i=0}^{s^{2}}I_{i}^{tar}\underset{c\in classes}{\sum }{\left(P_{i}\left(c\right)-\hat{P_{i}}\left(c\right)\right)}^{2}$$

where $\hat{P_{i}}$ in the Equation (9) is denoted as predicted value, $P_{i}$ is expressed as the training tag value. Figure 6 below shows a graph of the YOLO loss function node in TensorBoard; the input element were the loss output of conv2d_59, conv2d_67, and conv2d_75; where the input_1, input_2, and input_3 correspond to the gprErr, viouErr, and clsErr in Equation (6) respectively.

**Figure 7:**
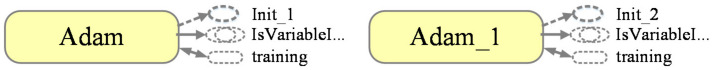
Adam optimizer in TensorBoard.

When using the gradient descent method to optimize YOLO v3 loss value, even though the loss function have to be optimized near the minimum value, there still exists a large gradient. In this way, using a global learning rate will cause some serious problems, such as slow gradient convergence or unstable loss value. In order to solve this problem, this article uses the Adam algorithm which is a learning rate adaptive algorithm improved by the RMSProp algorithm proposed by Kingma in 2014 [39]. First, we set a default learning rate (0.001 in TensorFlow) and two exponential decay rates for moment estimation (default is 0.9 and 0.990 in TensorFlow); then initialize the moment variable and its time step count; finally, we continuously correct the deviation through biased moment estimation to update the weight and learning rate. Figure 7 below shows two structural diagrams of Adam optimizers in TensorBoard.

## 3. Results and Discussion

### 3.1. Experimental Parameters

GPR model in this paper used the GX750-HDR (GEO AB Company, Sundbyberg, Sweden) of Swedish Guideline GEO AB Company. Sampling number collected for each channel was 412, sampling interval was 0.015 m, the coupling distance of GPR antenna preset was 0.14 m, and the diameter of the ranging wheel preset was 17 cm. GPR data preprocessing software was the REFLXW 7.5 which its copyright by K.J. Sandmeier. The training data set format adopted the COCO data format [40,41]. Here, we marked GPR image target for YOLO v3 training by the visual object tagging tool (VoTT) 2.1.0. Operating system was windows 10, and its processor model is Intel(R) Xeon (R) Gold 6130 CPU (Intel, Santa Clara, CA, USA) 2.10 GHz. Deep learning frameworks or related packages include the python 3.7, Keras 2.31, Tensorflow 1.13.1, cuDNN 7.4, Ananconda 3, Sklearn and GUDA 10.0. The main methods of preprocessing noise are: (1) Remove DC drift, (2) static correction cut, (3) gain, (4) remove direct ground wave, (5) remove high and low frequency signals, (6) horizontal smoothing. A total of 331 GPR image samples were collected in the experiment, of which the proportion of training set in whole data set is 70%, the validation set is 20%, and the test set is 10% in whole data [42]. In the YOLO v3 training stage, the batch size and subdivision of training sets are preset as 20. Epoch of each stage is preset as 51 and the learning rate is predefined as 0.001.

### 3.2. Anchor Boxes Selection by K-Means Clustering

**Figure 8:**
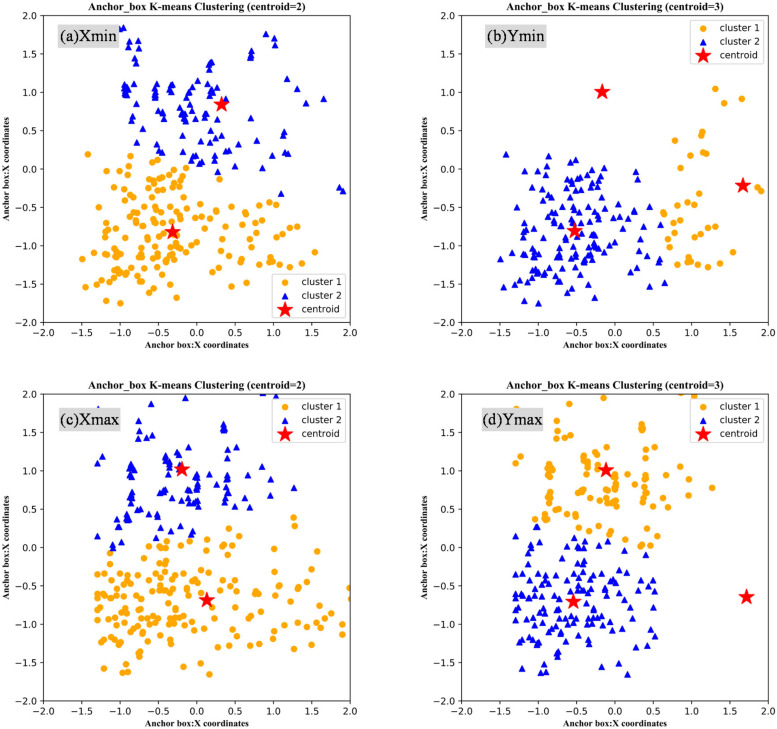
K-means clustering analysis of anchor boxes: (**a**,**b**) are related to Xmin and Ymin coordinates; (**c**,**d**) are related to Xmax and Ymax coordinates).

After using VOTT tool to label all hyperbola targets from GPR images, there are 386 rectangular boxes containing parabola generated from the training dataset of ground truth images. Location parameters of ground truth box are composed of four corner coordinates of the rectangular box as (x_min_, y_max_), (x_min_, y_min_), (x_max_, y_max_), and (x_max_,y_min_). Obviously, we only need to take four parameters x_min_, y_min_, x_max_, and y_max_ for clustering effect or silhouette coefficient analysis [43]. Silhouette coefficient is a significant evaluation index for clustering performance. Its value is commonly between [−1, 1]. When the silhouette coefficient is closer to 1, the cohesion and separation of K-means model are better. In Figure 8a, we adjusted the clustering or centroid number of K-means to 2; the maximum number of iterations is predefined as 200; after normalizing the x_min_, y_min_ data, it can be seen that the clustering group of centroid were still relatively demonstrable. The silhouette coefficient output by the silhouette score function from sklearn module was 0.4839. Compared with Figure 8b, when the number of centroid was set to 3, there exist high-separation and low-cohesive phenomenon for the clustered groups after standardized data. Similarly, Figure 8c,d shows the clustering effect of x_max_ and y_max_ data when the clustering is set to 2 and 3. At this time, the silhouette coefficient output by the silhouette score function was 0.4868. After calculation, finally we got four anchor boxes values for training configuration parameters that consist of x_min_, y_min_, x_max_, and y_max_.

### 3.3. V-IoU and NMS Training Loss Performance

**Figure 9:**
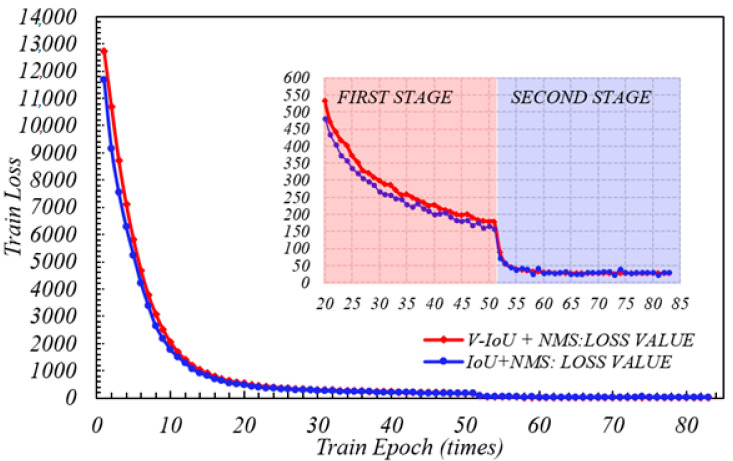
V-IoU versus IoU training loss (epoch: 0 to 83).

**Figure 10:**
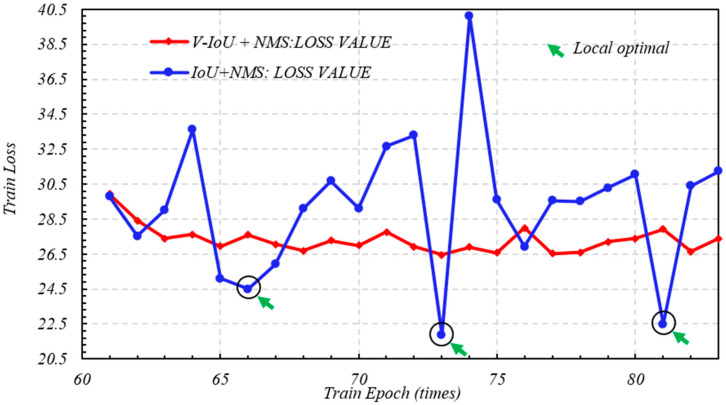
V-IoU versus IoU training loss (epoch: 60 to 83).

After derivation of Section 2.5, IoU-YOLO v3 loss function contains three parts. The first part is the average error loss of the centroid position in GPR bounding boxes which is centroid position ($t_{x}$,$t_{y}$) relative to ground truth boxes. Here, the coordinate related to x axis of the predicted bounding box can be denoted as$\;\hat{b_{x}}$ which is equal to$\;\mathrm{sigmoid}\;\left(t_{x}\right)+C_{x}\;$ and its coordinates related to y axis can be denoted as $\hat{\;b_{y}}\;\mathrm{which}\;\mathrm{is}\;\mathrm{equal}\;\mathrm{to}\;\mathrm{sigmoid}\;\left(t_{y}\right)+C_{y}$. Obviously after weight processing, the smaller the loss value, the closer the centroid between the predicted coordinate $\left(\hat{b_{x}},\hat{b_{y}}\right)\;$ and the true value$\;\left(b_{x},b_{y}\right)$, the better the prediction performance of logical regression function. In the first training phase of YOLO V3 with the V-IoU and NMS, when the epoch was less than 10, the loss value began to decrease very fast. When in the second stage, the convergence speed of loss function became steady and slow. Comparing the blue curve without adding V-IoU in Figure 9, the training performance of YOLO loss function seemed equivalent in two stages, but the completion time of entire 83 epochs was 3 h and 57 min. This is because the local optimization produced by the training process will affect the algorithm calculation efficiency to update function weights by back propagation. For this reason, as can be seen from Figure 10, the loss value of IoU + NMS had been changing back and forward between 22.5 and 40, and three local optimal solutions that appear at the positions are indicated by five green arrows; however, V-IoU + NMS was relatively stable, and it is undemanding to perform global gradient descent to find the global optimal solution. In order to prevent data over fitting, the loss function is considered to be sufficiently convergent; when the epoch was equal to 83 iteration was stopped.

### 3.4. YOLO v3 Detection Effect

**Figure 11:**
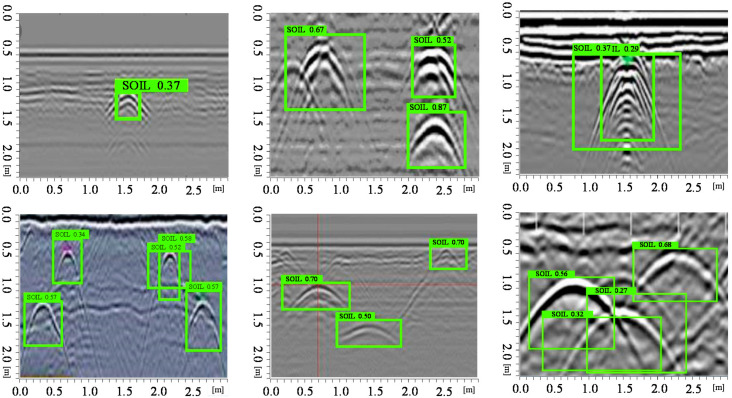
Single class targets detection performance

**Figure 12:**
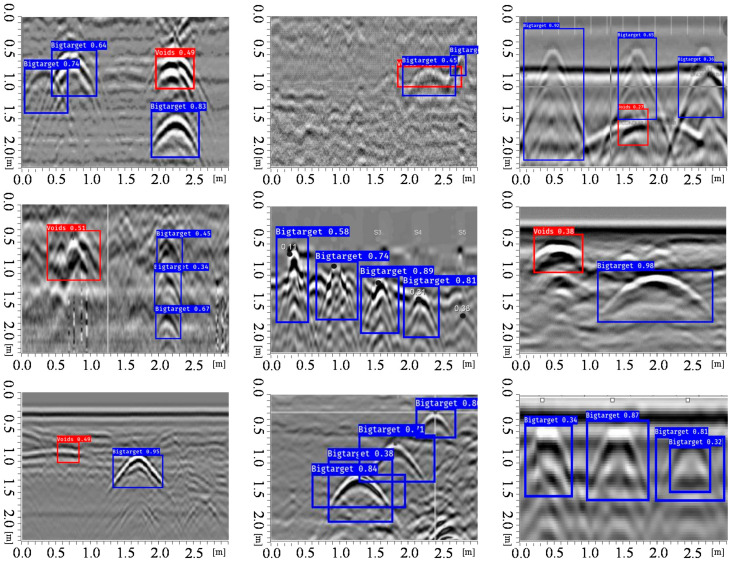
Multi-class class targets detection performance.

**Figure 13:**
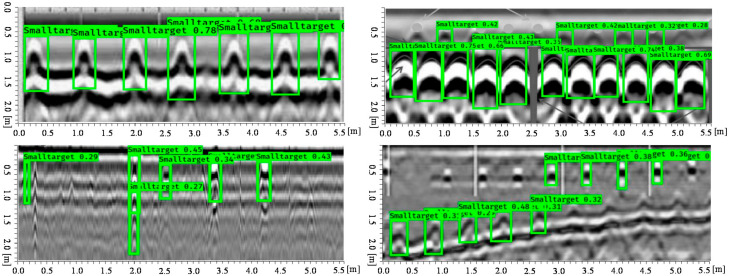
Pattern recognition of densely distributed reinforced concrete structures.

It can be known from the YOLO v3 network architecture in Section 2.1 that YOLO v3 can be detected on three feature maps of different scales and output after the input image size have been down sampled to 32, 16, and 8. Testing datasets contain three scenes for the real-time detected performance test, which cover the single class and multi-class pattern-recognition which include hyperbolic and voids features. The evaluation index refers the mean average precision (mAP) to training batches [44,45]. Assuming that P is denoted as the actual number of samples among target prediction, this is called precision. R is the recall rate, T is denoted as true positives, where$\;P=\frac{TP}{\left(TP+FP\right)}\;$ and $\;R=\frac{TP}{\left(TP+FP\right)}$, where TP is the true positives and FP the false negatives; the mAP can be calculated by equation$\;\frac{\sum AP}{N_{classes}}$, where AP is denoted as the average precision. Figure 11 shows the improved detection effect of YOLO v3 with V-IoU on single class targets. The verified data set showed in Figure 11, Figure 12 and Figure 13 were collected from the Soils research key Laboratory of South China Agricultural University. First, we detect the object’s physical position through GPR, and then mark the hyperbola vertex by the marking button on the MALA GPR controller. Finally, we use the difference between the identified rectangle midpoint and the marker’s value to determine the ground truth. Here, the V-IoU threshold was preset to 0.50. As can be seen from the figure, although some targets are small in the GPR image, the YOLO v3 detector can recognize it. This is because compared to YOLO v2, the V3 version has three detections, which are one down-sampled 13 × 13 and two up-sampled with 26 × 26, 52 × 52 feature maps. In addition, YOLO v3 have added a series of convolutional layer with 3 × 3 or 1 × 1 size that increase appropriately the number of channels. Overall, in this situation, total 132 hyperbolas in GPR image were tested. The correct detection number is 121, missed targets number is 7, and false alarm number is 10.

When there were multi-class targets in the detected GPR image, the predicted boxes can distinguish or identify the parabolas or voids. For some parabolas with multiple overlapping signals the vertex of curve was well positioned, as shown in Figure 12. Obviously, the less electromagnetic interference or noise in the GPR image, the better recognition and location performance. Those targets that are shallow from the soil surface had relatively obvious higher recognition scores. It can be seen that there were no misidentified boxes, all targets can be identified and located to the parabolic midpoint at overlapping positions. Figure 12 showed that the parabola with signal oscillation due to some highly conductive targets can be identified and located by the YOLO v3 detector with V-IoU. Overall, in this multi-class targets situation, total 82 hyperbolas in GPR image were tested. The correct detection number is 62, missed targets number is 4, and false alarm number is 5.

In engineering applications, we often need to detect the number of metal bars among concrete structures. It can be seen from Figure 13 that for the number of single-layer steel bars, the predicted bounding boxes can be positioned accurately; but for the multi-layer-reinforced concrete structure, there exists a case of missing identification. After many experiments and data statistics, if taking the number of hyperbola as a performance index, the YOLO V3 artificial intelligence recognition method proposed in this paper can predict the number of ground truth targets in GPR image by 90% accuracy, and its position error is less than 10% length unit. When detecting the number of concrete structures, total 192 hyperbolas in GPR image were tested. The correct detection number is 175, missed targets number is 11, and false alarm number is 8. Overall, YOLO v3 can achieve satisfactory performance when recognizing and positioning electromagnetic wave from GPR image features.

### 3.5. Learning Rate and Mean Average Precision Comparison

**Figure 14:**
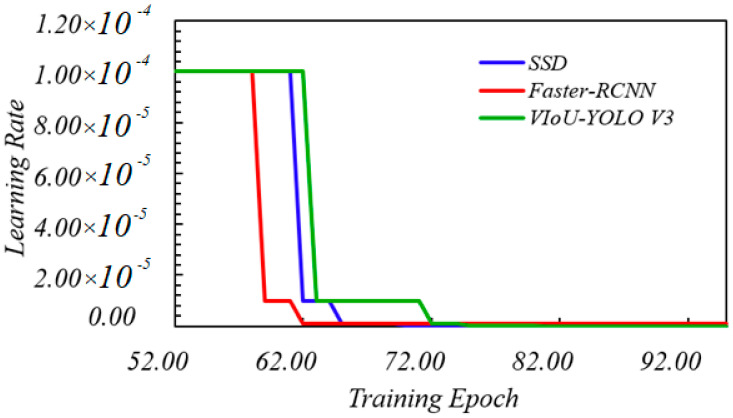
Learning rate versus training epoch.

**Figure 15:**
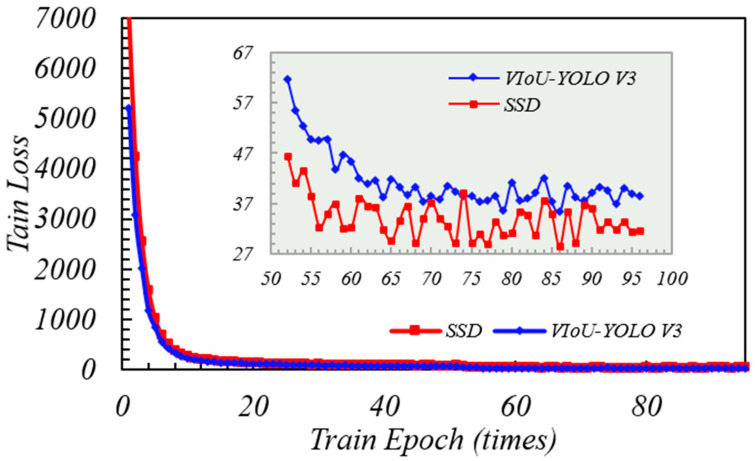
Loss versus training epoch.

The learning rate directly affects the convergence state of the YOLO v3 training performance, and batch size affects the generalization performance. Earlier, we have discussed the Adam adaptive algorithm to update the global learning rate. In TensorFlow, we set the initial parameters of the learning rate to the same value. Here we evaluate the model optimization of SSD, faster-rcnn, and VIoU-YOLO v3 through the change of learning rate in training epoch. As shown in Figure 14, the learning rates of SSD, faster-rcnn, and VIoU-YOLO v3 were between 52 and 72 in epoch. The YOLO v3 has converged to a stable value when epoch was 73, which made the updated weight of loss value in TensorFlow to be reduced to the global threshold in a shorter time. The change of SSD is very close to VIoU-YOLO v3, but we can see from Figure 15 that the same situation occurs again similarly to Figure 9. Loss value of the SSD model will easily converge to its local optimal value with the increase of training times; obviously, after comparing the learning rate and loss value, the convergent speed of YOLO v3 with VIoU is more ideal.

Furthermore, we compared mAP of SSD, faster-rcnn, YOLO v2 and YOLO v3 with different V-IoU (or IoU) thresholds and scenes. We used 300 GPR image samples to generate Table 1. Here, the mAP_50_ means its IoU threshold preset as 0.5 and mAP_75_ preset as 0.75. Similarly, the mAP_sc_, mAP_mc_, and mAP_metal_bars_ represents the single classification, multi-class targets detection and only contains single layer metal bars scenes respectively. As shown in Table 1, after comparison, when the V-IoU threshold was 0.50, YOLO v3 with darknet-53 as the backbone can achieve a maximum mAP of 83.16; the SSD with ResNet-34 as the backbone can achieve an mAP of 75.66. The mAP scores of Faster-RCNN, YOLO v2, and v3 are more or less. When the IoU threshold was 0.75, the mAP scores of YOLO v3 and VIoU YOLO v3 are 77.15 and 75.90, respectively; SSD achieved a maximum mAP score of 79.80. In the detection which have multi-classes targets of GPR image, it is clear that YOLO v3 achieved an ideal mAP score. Comparing the mAP score of single class scenes, V-IoU YOLO v3 scored 83.17; in addition, when detecting the metal bars underground, although YOLO v3 achieved the highest mAP score of 79.90, V-IoU YOLO v3 still scored 76.10. In general, V-IoU YOLO v3 can achieve the best performance for three different real-time scenes.

### 3.6. Real-Time Performance and fps Testing

**Figure 16:**
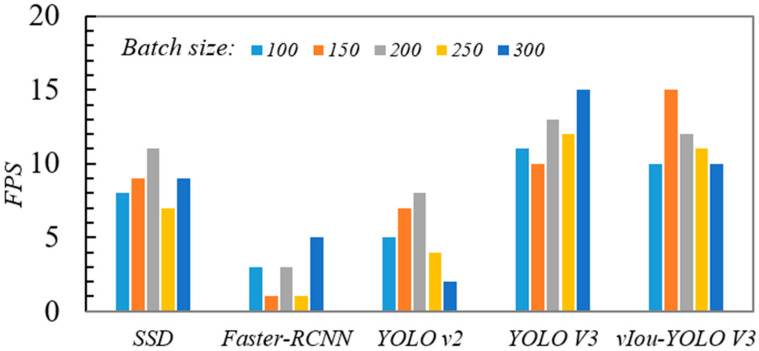
The fps versus batch size in five detection algorithms.

In expectation of testing the real-time detection speed of YOLO v3, we randomly selected five batches from 331 GPR images with size 416 × 416; the number of image batches were 100, 150, 200, 250, and 300, respectively, and took the mAP value in Table 1 as reference. Computer processor still is Intel(R) Gold 6130 with CPU with 2.10 GHz. As shown in Figure 16, when the batch size was 200, the detection speed of SSD can reach to 11 fps. After comparison, the detection speed of Faster-RCNN in each batch was not ideal, and its maximum detection speed is just 5fps. It can be seen from Figure 16 that the average detection speed of YOLO v2 is 5 fps. The fastest detection speed of YOLO v3 and VIoU-OLO v3 can reach 15fps, and their average value is around 12fps. In other words, when the vehicle is equipped with GPR device, its detection speed can reach between 10 km/h and 20 km/h. Consequently, the VIoU-YOLO v3 detection method proposed in this paper can fulfill the real-time detection requirements.

## 4. Conclusions

In this paper, a YOLO v3 was applied to build neural network detector to achieve real-time pattern-recognition of GPR images. It can be applied to actual underground detection engineering with meaningful accuracy and robustness based on Tensorflow, but this article is also limited to less samples and detection types of targets. Overall, this paper developed an innovative research application based on artificial intelligence algorithm in the field of electromagnetic wave detection. The main conclusions are as follows:

(1) Redefined the encode approach of YOLO v3 and proposed a labeling technique with using parabolic vertices as feature points; this provides a high-precision encoding technique for locating targets in GPR image.
(2) Proposed the principle of V-IoU; when the position of parabola vertex is within a certain range, free the limitation of IoU threshold. This method effectively reduces the false recognition rate caused by electromagnetic interference.
(3) The V-IoU-YOLO v3 neural network can achieve 83.17 mAP score in the single class pattern-recognition scenes and 76.10 mAP score when detecting metal bars in concrete structures.
(4) The VIoU-YOLO v3 detecting speed can reach 15fps under the CPU processor, and this speed can meet the real-time operation requirements of vehicle equipped with GPR device.

## Figures and Tables

**Table 1:** mAP comparison with five detection algorithms.

Algorithm	Backbone	mAP_50_	mAP_75_	mAP_sc_	mAP_mc_	mAP_metal_bars_
SSD	ResNet-34	75.66	79.80	79.37	71.44	66.31
Faster-RCNN	ResNet-18	81.45	66.22	74.21	77.09	68.51
YOLO v2	Darknet-19	80.34	72.05	80.08	66.15	68.92
YOLO v3	Darknet-53	83.16	77.15	85.82	76.30	79.90
VIoU-YOLO v3	Darknet-53	82.71	75.90	84.56	83.17	76.10
